# Evaluation of the Effects of the Quaternary Ammonium Silane K21 on Zebrafish Viability, Toxicity, Growth, and Development

**DOI:** 10.3390/biomedicines13061267

**Published:** 2025-05-22

**Authors:** Surendra K. Rajpurohit, Devan Anmol S. Manhiani, Ashwin Ajith, Pragya Rajpurohit, Simran Hotwani, Sai Nasanally, Arsha Sreekumar, Keshu Bhat, Aiden Van Derhei, Rohan Pasi, Arishia Mishra, Kirk Kimmerling, Clifton M. Carey

**Affiliations:** 1 Georgia Cancer Center, Medical College of Georgia, Augusta University, Augusta, GA 30912, USA; 2 College of Pharmacy, Medical University of South Carolina, Charleston, SC 29425, USA; pragyaraj2018@gmail.com; 3 FiteBac Technology, Marietta, GA 30064, USA; kirkkimmerling@fitebac.com; 4 School of Dental Medicine, University of Colorado, Denver, CO 80309, USA; tranquil4007@gmail.com

**Keywords:** zebrafish, experimental therapeutics, quaternary ammonium silane K21, viability, toxicity, development, growth, in vivo study

## Abstract

**Background**: The FDA-cleared antimicrobial quaternary ammonium silane K21 is recognized for its antimicrobial properties. This study explored potential applications of the K21 molecule in human health protection, disease prevention, and treatment using the zebrafish model. **Method**: A multi-dimensional approach was utilized to assess the toxicity, tolerance, and optimal dosage of K21 through serial dilutions at various concentrations. Acute and chronic exposure studies were performed at different developmental stages (embryonic, larval, juvenile, and adult) to evaluate its efficacy and toxicity in wild-type (WT), Casper (transparent skin mutant), and transgenic zebrafish lines. **Results**: Significant weight gain was observed in the F1 generation following K21 treatment, a trend that continued into the F2 and F3 generations. The effects of K21 on lipopolysaccharide-induced inflammation were also examined in Casper NFkB:GFP transgenic lines. Treatment with K21 reduced inflammation, indicating anti-inflammatory properties. Improved hatching rates, accelerated larval development, an increased adult mass, and modest reductions in embryonic motility (less than 20%) suggested positive developmental influences. Single-cell RNA sequencing further validated the biological impacts of K21, revealing the potential activation of a novel pathway that accelerates zebrafish growth. **Summary and Conclusions**: These findings position K21 as a promising candidate for biomedical applications and aquaculture, warranting further investigation into its underlying molecular mechanisms. Our additional study on the effect of K21 on the artemia (brine shrimp) hatching process provide strong evidence of better hatching ratio of 90% for brine shrimp in the group with K21 drug treatment as compared to 70% in the group without the K21 drug at 24 h of treatment; the K21 drug helps the early hatching process, as observed the 90% hatching rate in 20 h K21 treatment group hatching while in the group without K21, only 40% of brine shrimps hatched.

## 1. Introduction

Zebrafish (*Danio rerio*) is a frequently used vertebrate model in biomedical research because of its genetic homology with humans, ease of maintenance, and high fecundity. Approximately 70% of human genes have at least one zebrafish ortholog, and nearly 82% of human disease-related genes are conserved in zebrafish [1]. These characteristics render zebrafish a powerful model for studying human diseases and therapeutic interventions [2]. Its transparent embryos allow real-time imaging of vascular, neural, and organ development, facilitating high-throughput screening in drug discovery, toxicology, and regenerative medicine [3].

Apart from its applications in human disease modeling [4], zebrafish is also extensively used in pharmacogenomics [5]. The rapid developmental cycle of zebrafish and its external fertilization enable large-scale screens of small molecules [6,7]. The ability to monitor physiological responses at the whole-organism level makes zebrafish an ideal model for evaluating toxicity, drug efficacy and metabolic responses [8,9] and for high-throughput drug discovery [10,11]. In addition, the zebrafish genome may be easily manipulated using transgenic lines [12,13]. This asset enables the study of specific molecular pathways underlying physiological and pathological conditions.

The quaternary ammonium silane compound codenamed K21 is a US Food and Drug Administration-cleared antimicrobial agent [14,15]. During the authors’ toxicity studies in zebrafish, exposure to K21 serendipitously resulted in enhanced growth without alterations in bone density. To understand the molecular mechanisms behind this effect, single-cell RNA sequencing was conducted on zebrafish larvae grown in K21-treated water. The major upregulated genes included those involved in oxygen-binding, photoreceptor activity, and G-protein-coupled receptor signaling. These findings suggest that K21-treated zebrafish have enhanced visual function that results in improved prey detection and feeding behavior, possibly contributing to an increased body mass [16]. Because of the correlation between retinal function and metabolic disorders such as diabetes [17], these findings highlight a potential therapeutic role for K21 in diabetic retinopathy and retinal neuroprotection. Moreover, the observed accelerated growth raises the possibility of using K21 in aquaculture to enhance fish development and optimize feeding efficacy.

**Figure 1:**
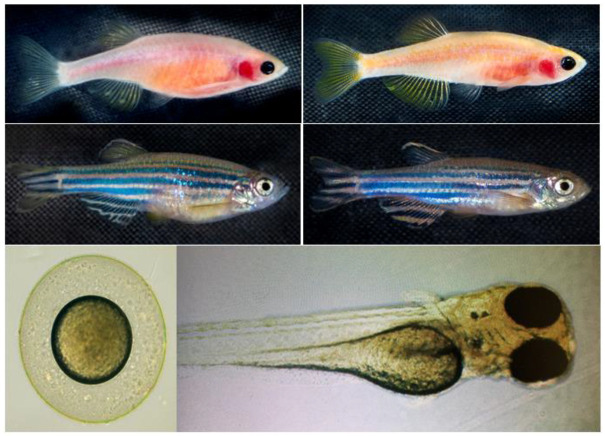
Zebrafish phenotypes used in this study. Representative images of the zebrafish phenotypes used in the experiment. The Casper (roy^−^/^−^, nacre^−^/^−^) strain (top row) lacks melanophores and iridophores, resulting in a transparent body, whereas the wild-type AB (WT-AB) strain (bottom row) exhibits the characteristic pigmented and striped appearance. Each phenotype includes both female (left column) and male (right column) specimens. The developmental stages of zebrafish: the lower left panel represents embryonic stage, and lower right panel represents the larval stage. These distinct phenotypic backgrounds were utilized to assess the effects of K21 treatment on growth, development, and physiological responses.

The objective of this study was to systematically evaluate the physiological and molecular effects of K21 on zebrafish development across multiple generations. This included assessing growth patterns, metabolic responses, and neural activity in wild-type, Casper (roy^−^/^−^, nacre^−^/^−^), and transgenic zebrafish lines (Figure 1). Casper is a transparent zebrafish double mutant strain that lacks iridophores (roy^−^/^−^) and melanophores (nacre1/1). It is named after the cartoon ghost “Casper” because of its see-through body, which allows for the real-time visualization of internal organs, tissues, and cellular processes without the need for invasive procedures. The overall hypothesis tested was that K21 exposure enhances zebrafish growth and development through the modulation of metabolic and neuronal pathways, resulting in increased feeding efficiency and overall body mass.

## 2. Materials and Methods

### 2.1. Preparation of System Water

The zebrafish facility water system was operated using a Zebrafish System Water Unit to ensure a continuous and controlled aquatic environment for zebrafish maintenance [18]. The system was designed to provide 400 gallons of reverse osmosis water storage to enable a 10% daily replacement to maintain water quality and stability. Water parameters were regulated through automated sensor systems. pH levels were maintained by the automated injection of a sodium bicarbonate solution, while conductivity was controlled through the automated injection of a sea salt solution. Temperature regulation was integrated into the built-in water heater system of the Zebrafish System Water Unit. To ensure water cleanliness, the system incorporated charcoal filtration and routine filter replacements. Hygiene was maintained through an ultraviolet sterilization system integrated within the Zebrafish System Water Unit to provide a stable and pathogen-free environment for zebrafish housing and breeding.

### 2.2. Zebrafish Housing

Zebrafish were raised and maintained at the Transgenic Zebrafish Core Facility of Augusta University. This facility provides a controlled environment to ensure optimal biological conditions, including strict water sterility and advanced filtration systems that regulate pH, temperature, conductivity, and ultraviolet light sterilization. A consistent 14:10 h light–dark cycle was maintained, and the water temperature was kept between 27.0 and 28.5 °C to support the proper development of embryos, larvae, and adult zebrafish.

Zebrafish strains were housed in appropriately sized tanks based on their developmental stage and population density to ensure optimal welfare and experimental reproducibility.

All experimental procedures involving zebrafish were conducted in accordance with the approved protocol standards set by the Institutional Animal Care and Use Committee at Augusta University, Augusta, Georgia. These protocols ensured that ethical guidelines and regulatory compliance were strictly followed in all aspects of zebrafish care, handling, and experimental procedures.

### 2.3. Zebrafish Strain and Progenies

Different strains of zebrafish progenies were generated, grown, maintained, and utilized for experimental purposes. Zebrafish were used at all developmental stages, including embryo, larval, juvenile, and adult phases. The zebrafish lines included throughout the project were: wild-type AB, Casper (*roy^−^/^−^, nacre^−^/^−^*) [19], Tg (*myl7:RFP;secA5:YFP*) [20], Tg (*Fli:GFP*) [21], Tg (*mpeg:mCherryFP*) [22], Casper; Tg (*myl7:RFP;secA5:YFP*) [23] Casper; Tg (*Fli:GFP*), Casper; Tg (*mpeg1:mCherryFP*) unpublished data], Casper; Tg (*NF-kB:GFP*) [24], and Casper; Tg (*myl7:RFP;secA5:YFP; Fli:GFP*) unpublished data] (Figure 1).

### 2.4. Breeding, Embryo Collection, and Larval Screening

On day 0, male and female zebrafish from selected strains were paired as breeders and placed into breeding tanks to facilitate mating. On day 1 (0–24 h post-fertilization (hpf)), embryos were collected from the bottom of the breeding tanks and unhatched eggs were removed. After removing the debris, the embryos were transferred to a clean hatching tank for further development. At 12 hpf, the embryos were cleaned, and dead embryos were removed. Viable embryos were treated with 1-phenyl-2-thiourea (PTU, 0.003% or 200 µM) to inhibit pigmentation. By day 3 (49–72 hpf), embryos hatched into larvae, which were screened for transgene expression using fluorescence microscopy. The larvae were used at same age for the genomic assay and flow cytometry assay. Transgenic and non-transgenic zebrafish progeny were separated, and each group was transferred to fresh system water to facilitate their development into adulthood and maintain colonies across multiple generations.

To inhibit pigmentation, a 50× stock solution of 1-phenyl-2-thiourea (PTU) was prepared by dissolving the compound (1.520 g) in 1 L of distilled water. The mixture was stirred on a hotplate at 50 °C for at least 2 h while protected from light. After cooling to room temperature, the stock solution was aliquoted and stored at −20 °C until use. To prepare the 1× working PTU solution, the 50× stock solution was diluted with fresh system water to achieve the desired concentration for experiments.

### 2.5. Acute Exposure and Toxicity

Stock solutions and working medium of K21 were prepared using a stepwise dilution process. K21 Stock-1 was supplied by Largent Health LLC (Marietta, GA, USA). K21 Stock-2 was prepared by mixing 10 µL of K21 Stock-1 with 190 µL of absolute ethanol, yielding a total volume of 200 µL K21 Stock-3 was then prepared by diluting 200 µL of K21 Stock-2 in 19.8 mL of ZF system water, resulting in a total volume of 20 mL. Finally, the K21 working medium was prepared by adding 1 mL of K21 Stock-3 to 999 mL of zebrafish system water, producing a total volume of 1000 mL (0.001 ppm/L). The optimization of K21 dose was further cross validated using 0.5% of K21 (K21 QAS H2) in water, with a final concentration of 0.001 ppm/L (1 µg/L). In this way, the final concentration of K21 was established as the optimal dose in the range of 0.001 ppm of K21 per liter of ZF system water.

The K21 working medium was mixed with fish system water at an initial 1000 µg/100 mL concentration. The mixture was then serially diluted to achieve final concentrations of 500 µg/100 mL, 250 µg/100 mL, 125 µg/100 mL, 62.5 µg/100 mL, and 31.25 µg/100 mL in fish system water. If signs of toxicity were observed at the initial concentration ranges, additional serial dilutions were performed in sets of six progressively lower concentrations. This process continued until a concentration range was identified where no signs of toxicity were present. The non-toxic concentration range was designated as the reference range for subsequent studies.

Once the concentrations were prepared, six adult zebrafish were placed into each group, with one fish per cup containing system water and the corresponding concentration of K21. A negative control group, consisting of only system water, was included in the experiment, with six fish in each set. This resulted in a total of 36 fish per concentration range (5 + 1 concentrations × 6 fish). The maximum number of dilution cohorts was expected to be three, resulting in a total of 108 fish (3 rounds of 36 fish).

The zebrafish were monitored while remaining in the system for 24 h. If any fish exhibited obvious signs of distress (i.e., hyperactivity, physical injuries, lesions, unusual swimming patterns, or settling), they were removed and euthanized. Death due to acute toxicity was also recorded. The experimental endpoint was defined as a concentration range in which no zebrafish exhibited distress. At the conclusion of the study, the remaining fish were euthanized after determining the reference dosage.

This experimental protocol was applied to adult zebrafish, as well as those in the embryonic (day 0), larval (day 3), and juvenile (day 15) stages. The total number of zebrafish used for this experiment was 432 (108 fish per experiment × 4 age points).

### 2.6. Development of a Chronic Exposure Timeframe

Using the reference dosage determined in Section 2.5, a single fish was placed in a cup containing system water with the designated drug concentration. Exposure occurred over time intervals of 2, 4, 7, and 14 days. Six fish were used at each time point and concentration level, resulting in a total of 120 fish (6 fish × 5 concentrations × 4 time points).

When all fish in the initial reference concentration exhibited signs of distress at the 2-day exposure, the concentration range was further diluted at a 1:2 ratio, and the experiment was repeated with the lower concentration. This process continued until a concentration range was identified in which all fish remained viable for the full 14-day period. This established the final exposure and concentration range for subsequent studies. The use of three dilution cohorts resulted in using 360 fish in this phase of the study (120 fish/range × 3 ranges).The fish were monitored twice daily for signs of distress, including hyperactivity, physical injuries or lesions, abnormal swimming behavior, or settling. The affected fish were euthanized if distress was observed. Death due to acute toxicity was also recorded.

This experimental protocol was repeated for zebrafish at different developmental stages, including embryos (day 3), larvae (day 7), juveniles (day 15), and allowed to the adult stage (three-month-old). At the conclusion of the study, all adult fish were measured and weighed before being paired for breeding the next generation. By keeping the fish progeny in the final concentration of K21 medium in fish system water (1 μL per L), the fishes at earlier developmental stages were allowed to mature into adults before undergoing measurement and selection as breeders for subsequent generations in K21 medium.

### 2.7. Effect of Chronic Exposure of Zebrafish to K21 over Multiple Generations

The K21-treated zebrafish and their negative controls were paired for breeding at the conclusion of experiment described in Section 2.6. These zebrafish were maintained in either K21 + system water or in system water alone as the control group. Their offspring were raised under the same respective conditions and allowed to develop into adults.

The zebrafish were weighed upon reaching adulthood and their lengths were measured. The fish were then sorted into breeding pairs based on size within their respective groups to produce the next generation. The same experimental process was repeated for the subsequent generation. Fifteen breeding pairs produced 1000 fish for the F1 generation. These F1 zebrafish were then bred to generate an additional 1000 fish for the F2 generation. The procedure was repeated to generate another 1000 fish for the F3 generation.

### 2.8. The Study of the Efficacy of K21 Treatment on the Brine Shrimp Hatching Process

Brine shrimps, which serve to feed many different fish species in culture, have been commonly used in ecotoxicological testing because of their capacity to adapt to different environments. In the present study, brine shrimps (*Artemia* sp.) were used as the primary consumer, and zebrafish as the secondary consumer. We are conducting multiple research projects to study the K21 drug treatment for the zebrafish model system and evaluate K21 in health preservation and disease prevention using a zebrafish model and its environmental enrichment. Such landmark novel findings allow us to study the efficacy of K21 treatment on the brine shrimp hatching process and determine its potential role to betterment of the brine shrimp, which is a primary food recipe for zebrafish from larval to adult stages. In this study, the brine shrimps were exposed to K21 an environmentally relevant concentration (1 µg of K21 per liter of ZF system water).

### 2.9. Histological Examination

Adult zebrafish treated with K21 and control specimens were euthanized following institutional guidelines. The whole fish were immediately fixed with 4% paraformaldehyde in phosphate-buffered saline (PBS) at 4 °C for 24 h to preserve the tissue morphology. After fixation, specimens were washed with PBS and dehydrated through an ethanol gradient (50%, 70%, 80%, 90%, 95%, and 100%) before being cleared in xylene and embedded in paraffin wax.

Paraffin-embedded zebrafish were sectioned using a microtome (Leica RM2255, Leica Biosystems, Wetzlar, Germany) at a thickness of 5 µm. The sections were mounted onto glass slides (Superfrost Plus, Thermo Fisher Scientific, Waltham, MA, USA) and air-dried before deparaffinization in xylene and rehydration through a descending ethanol gradient. Sections were stained with hematoxylin and eosin (H&E) for histological examination.

Tissue sections from the anterior (heart region) and posterior (abdominal region) regions of K21-treated and control zebrafish were analyzed for structural integrity, cellular morphology, and any pathological changes. A comparative analysis between groups was performed to assess the impact of K21 treatment on organ and tissue development. Representative images of the heart and abdominal regions were selected to illustrate the observed histological differences.

### 2.10. Genetic and Phenotypic Analyses of the Impact of K21 Using Genetically Engineered Strains

Following the K21 studies in wild-type zebrafish, the experiments were repeated using eight genetically engineered strains:

- Casper (transparent skin mutant);
- Casper *myl7:RFP; A5:YSP* transgenic line for cardiac function;
- Casper *NFkB:GFP* transgenic line for inflammation;
- Casper *mpeg:mCherryFP* transgenic line for CNS microglial function;
- Casper *Fli:GFP* transgenic line for the vasculature;
- Wild-type WT:AB strain, used as the control.

Confocal laser scanning microscopy (Stellaris-5, Leica Microsystems GmbH, Wetzler, Germany) was performed in addition to length and weight measurements. Imaging of the zebrafish was conducted at the 72 hpf stage to evaluate transgene expression. Imaging was performed for all transgenic strains and included both the initially exposed generation and subsequent generations under chronic exposure. Images were captured and analyzed using LAS-X software (Version 4.6.1.27508; Leica Microsystems, Belgrade, Serbia).

At this stage, the zebrafish larvae were immobile and did not require anesthesia. The larvae were transferred to a cell imaging facility for fluorescent and confocal microscopy. During this process, they were sorted based on fluorescent transgene expression before returning to the laboratory for further growth. Fluorescence screening was conducted only on larvae that remained in the fish water system to ensure consistency in the experimental conditions. A total of 23,232 zebrafish were used in this experiment (3872 fish per strain for six transgenic lines). A subset of these zebrafish was also maintained as a small breeding colony for future studies.

### 2.11. Lipopolysaccharide-Induced Inflammation Model in a Transgenic Zebrafish Strain

Novel roles of nuclear factor kappa-light-chain-enhancer of activated B cells (NF-κB) signaling in endocrine differentiation and serotonergic signaling were identified using the Tg (6xNF-kB:EGFP); Casper (roy^−^/^−^, nacre^−^/^−^) strain of zebrafish developed by the senior author [24]. To validate the value of Casper Tg (NF-κB:GFP) homozygous strain in NF-κB signaling, experiments were conducted to assess its inflammatory response following bacterial lipopolysaccharide (LPS) exposure and to evaluate the inhibitory effects of both established and novel anti-inflammatory compounds.

Thioctic acid (α-lipoic acid) is recognized for its antioxidant properties and has been reported to inhibit NF-κB activation, thereby reducing inflammation [25]. Parthenolide, a sesquiterpene lactone derived from the medicinal herb feverfew (*Tanacetum parthenium*), inhibits NF-κB signaling [26]. The authors have previously validated the inhibitory effects of these two compounds on NF-κB activation using the Tg(NF-kB:GFP) transgenic zebrafish line [12]. In the present study, K21 was evaluated to determine whether it similarly possesses anti-inflammatory properties by inhibiting NF-κB activation.

Three-day-old larvae from both the transgenic strain and the control Casper (roy^−^/^−^, nacre^−^/^−^) strain were divided into multiple treatment groups as follows:

1. Control group—system water only;
2. LPS group—system water with 100 µg/mL LPS (*Escherichia coli* 0111:B4, MilliporeSigma, Burlington, MA, USA);
3. LPS + K21 group—system water with 100 µg/mL LPS and 10 µg/mL K21 working medium;
4. K21 only—system water with 10 µg/mL K21 working medium.

Each group consisted of 24 larvae. Each larva was placed in an individual well of a 96-well plate containing 300 µL of the respective treatment solution. The plates were covered to prevent evaporation and maintained at 28 °C for a 4-day treatment period. After the treatment period, the larvae were homogenized using a glass tissue grinder (Thermo Fisher Scientific, Waltham, MA, USA), and the homogenate was filtered through a 70 µm nylon mesh sterile cell strainer (Thermo Fisher Scientific). The resulting cell suspensions were analyzed using a Cytek Aurora Spectral Flow Cytometer (Cytek Biosciences, Fremont, CA, USA) to quantify NF-κB activation by measuring the fluorescence intensity of enhanced green fluorescent protein. Data acquisition and analysis were performed using the SpectroFlo^®^ software (Cytek Biosciences, V3.0).

### 2.12. Inhibition of Zebrafish Pigmentation for High-Resolution In Vivo Imaging

To enhance the detection of transgenic reporters during imaging and screening procedures, pigmentation was inhibited in zebrafish larvae with PTU to promote transparency until the assay was completed. Larvae were screened up to 72 hpf. At this stage, the larvae remained immobile, which eliminated the need for anesthetic agents. Fluorescence microscopy was performed at 72 hpf to confirm transgene expression. Imaging was conducted using a Keyence fluorescence microscope (model BZX-800, Keyence, Osaka, Japan). The fluorescence-based screening procedure enabled the precise detection of transgene expression while maintaining optimal imaging resolution.

### 2.13. In Vivo Time-Lapse Imaging

Confocal laser scanning microscopy (Stellaris-5) was used to observe cellular pathology in affected tissues. After imaging, the fish were returned to controlled environmental conditions. The acquired images were analyzed using ImageJ software (V 1.54p, National Institute of Health, Bethesda, MD, USA). Fluorescent signals were detected based on their specific excitation and emission wavelengths. Green fluorescent protein was excited at 488 nm with an emission wavelength of 507 nm, while red fluorescent protein (RFP) was excited at 558 nm with an emission wavelength of 583 nm.

### 2.14. Immobilization of Zebrafish Larvae for In Vivo Imaging

Zebrafish larvae remained non-motile until 72 hpf, as they had not yet developed the ability to swim [27]. After this stage (≥4 days old), the larvae became motile and began swimming. A clove oil-based anesthetic solution was used to prevent movement and minimize stress or pain during microscopy and imaging procedures [28]. A 0.02% clove oil working solution was freshly prepared by dissolving clove oil in zebrafish system water. This minimal concentration was used because of recent reports of eugenol on embryonic development and behavioral changes in the zebrafish [11,29,30]. The solution was incorporated into the working medium to immobilize the larvae. This procedure facilitated accurate screening and imaging using fluorescence and confocal laser scanning microscopy. Additional measures were implemented to further reduce stress during imaging, including dimmed lighting and minimized noise levels in the imaging environment.

### 2.15. Flow Cytometry

Flow cytometry was used to quantify NF-κB activation by measuring green fluorescent protein (GFP) expression in zebrafish larvae from the Casper Tg(NF-kB:GFP) transgenic line. Four-day-old larvae exposed to various experimental conditions—system water (control), lipopolysaccharide (LPS, 100 µg/mL), LPS combined with K21 (10 µg/mL), and K21 alone (10 µg/mL)—were individually homogenized using a glass homogenizer (Pyrex Potter tissue grinder; Corning Life Sciences, Tewksbury, MA, USA). Following homogenization, the cell suspensions were filtered using a sterile 70 µm nylon mesh cell strainer (Thermo Fisher Scientific) to obtain a single-cell suspension.

The single-cell suspensions were analyzed at the Augusta University Flow Cytometry Core Facility using a Cytek Aurora Spectral Flow Cytometer (Cytek Biosciences). Instrument calibration and compensation were performed using appropriate fluorescent controls, and fluorescent signals were detected and quantified using SpectroFlo^®^ software provided by Cytek Biosciences. The fluorescence intensity of GFP served as a marker for NF-κB activation levels to evaluate inflammatory responses among the experimental groups.

### 2.16. Genomics

Single-cell RNA sequencing (scRNA-seq) was conducted to evaluate the molecular impact of K21 treatment on zebrafish larvae. Larvae exposed to either K21 or the control medium were harvested, and viable cells were isolated using the EasySep™ Dead Cell Removal Kit (STEMCELL Technologies, Vancouver, BC, Canada). Approximately 14,000 live cells from each treatment group were loaded onto the Chromium Controller (10× Genomics) to generate single-cell RNA sequencing libraries using the Chromium Next GEM Single Cell 3′ Reagent Kit v3.1 (10× Genomics, Pleasanton, CA, USA) according to the manufacturer’s protocol. Libraries were sequenced on the Illumina NextSeq 500 system using a Mid Output v2.5 (150 cycles) kit, with 28 bp for Read 1, 8 bp for indexing, and 91 bp for RNA Read 2. This sequencing approach resulted in approximately 33,000 mean reads per cell. Raw sequencing reads were aligned to the zebrafish reference genome (GRCz11_93) using the STAR aligner integrated with the Cell Ranger pipeline (version 5.0.1; 10× Genomics), resulting in the identification of distinct cell population clusters and their corresponding marker genes.

Data preprocessing, normalization, differential gene expression analysis, and clustering were performed using the Seurat R package (v 4.4.3) [31]. Differentially expressed genes (DEGs) were identified based on statistical significance (adjusted *p*-value < 0.05) and log_2 fold change thresholds. For cluster annotation, the top DEGs within each cluster were identified based on the highest expression ratios relative to other cell clusters in the transcriptomic dataset. The expression profiles obtained for each cluster were further compared to the corresponding datasets available in public databases, such as the Zebrafish Information Network. Processed Seurat objects were converted into cloupe files using the LoupeR package (v 4.2.1) to facilitate visualization and detailed analysis in Loupe Browser (v8.0; 10× Genomics). Gene Set Enrichment Analysis (GSEA) was subsequently conducted to identify biological pathways that were activated or suppressed by K21 treatment. The results were visualized using volcano plots, dot plots, and bubble plots to highlight significantly altered genes and enriched pathways associated with K21 exposure.

### 2.17. Statistics

The statistical analysis was performed using GraphPad Prism 9 (v.9.0.0). Graphical data are presented as means ± SEMs, if not stated otherwise. If datasets followed a normal distribution and comparisons were performed between 2 experimental groups, then an unpaired, 2-tailed Student’s *t* test was used. *p* < 0.05 was considered statistically significant.

## 3. Results and Discussion

### 3.1. Optimization of the K21 Dosage for Zebrafish Aquatic Housing

Serial dilution titration was conducted to establish a standard K21 dose for zebrafish aquatic housing. Dilutions ranged from 100 µL/L to 1 µL/L. Exposure to 100 µL/L was determined to be lethal, as all zebrafish died within 1 h. At 10 µL/L, the fish survived beyond 24 h but exhibited signs of an overdose. A concentration of 2 µL/L, extended survival was observed for up to three days; however, it was still considered excessive. At 1 µL/L, zebrafish in the experimental group remained viable for over a week, maintaining normal functional and behavioral activities without signs of stress. Further exposure for up to one month was confirmed that vital activities remained intact. Accordingly, 1 µL/L was determined to be the optimal K21 concentration for zebrafish system water.

After establishing this concentration in adult zebrafish, the same standard dose was validated in eggs, embryos, larvae, and juveniles. Control groups were maintained in system water without K21, while the K21 experimental group was exposed to K21-treated water throughout their entire life cycle.

### 3.2. Zebrafish Development and Growth in K21-Treated and Untreated Control Populations

This part of the study evaluated the long-term, multi-generational effects of K21 exposure on a wild-type zebrafish strain under a standardized dosage regimen. Fish progenies from the F1-F3 generations were generated under K21 treatment. The major effects of K21 on zebrafish growth and developmental processes are summarized in Table 1.

**Figure 2:**
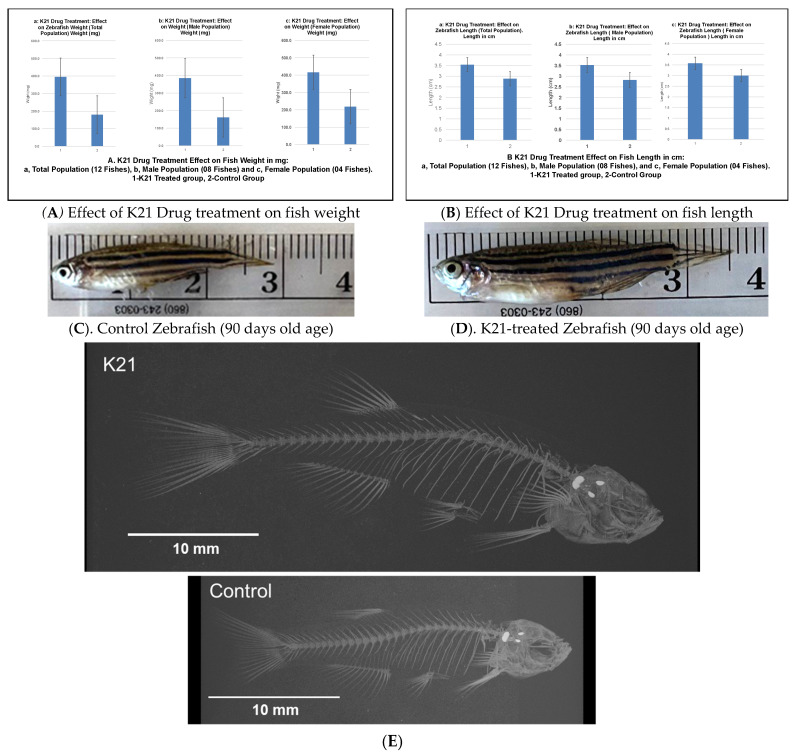
Effects of the K21 treatment on zebrafish growth and skeletal development. (**A**). Zebrafish treated with K21 exhibited a significantly increased body weight compared to the control group. Weight measurements were recorded for the total population (a), male population (b), and female population (c), showing consistent growth enhancement across sexes. (**B**–**D**). Fish length was also significantly greater in K21-treated zebrafish, with comparisons presented for the total population (a), males (b), and females (c). (**E**). The skeletal analysis of adult zebrafish revealed that K21-treated fish were significantly longer and taller than controls (*p* < 0.05), with the vertebral size being larger in the treated group (*p* < 0.001). No significant differences were observed in the vertebral bone density or rib width (*p* > 0.05), but the mineral density of the vertebrae was higher in K21-treated fish.

Figure 2A illustrates the impact of the K21 treatment on fish weight across the total population, as well as in male and female subpopulations. The K21-treated group exhibited a significant increase in mean weight compared to the control group, with statistical significance observed (*p* < 0.05). Male zebrafish in the K21-treated group showed a greater weight gain than their female counterparts.

Figure 2B presents the effect of K21 on fish length. Zebrafish exposed to K21 displayed a significant increase in length compared to the control group (*p* < 0.05). Similar to weight, male zebrafish demonstrated a greater increase in length than females. These findings suggest that K21 treatment positively influenced zebrafish growth, with notable improvements in both weight and length across multiple generations.

Figure 2C further supports the observation that K21 treatment enhances zebrafish growth. K21-treated adult fish were statistically longer in length than those in the control group (*p* < 0.05). Despite the increased size, no significant differences were observed in eye size, organ morphology, or pigmentation patterns. These observations indicate that K21 did not induce developmental abnormalities.

An analysis of the vertebral structure in 14 fish per group revealed that the average vertebral size was significantly larger in K21-treated fish compared to controls (*p* < 0.01). However, the vertebral bone density and overall fish body density were not significantly different between the groups (*p* > 0.05). The minimal density of the vertebrae was larger in K21-treated fish, suggesting a potential increase in overall skeletal robustness (Figure 2D). These findings indicate that K21 promotes overall zebrafish growth without compromising skeletal integrity or inducing malformations. Zebrafish exposed to K21 in their food have been observed to grow larger than control fish. It was unknown if the growth could have resulted in physical differences between the K21-exposed fish and the control fish (other than size) (Figure 2E).

### 3.3. Effect of K21 on Wild-Type Zebrafish Viability and Survival

**Figure 3:**
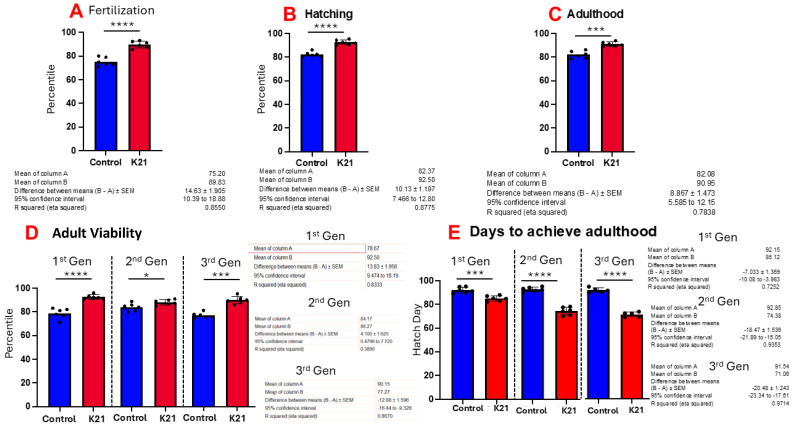
Effect of K21 on wild-type zebrafish viability and survival. (**A**) Effect of K21 on zebrafish egg fertilization; (**B**,**C**) effect of K21 on zebrafish hatching and growth at the embryonic, larval and adult stages. (**D**) Effect of K21 on the overall survival of fish from the first, second and third generations. (**E**) Effect of K21 on the achievement of the adulthood timeline in the first, second and third generations of zebrafish. The graph represents six technical replicates (n = 6/group, means ± SEMs, * *p* < 0.05, *** *p* < 0.001, **** *p* < 0.0001).

Figure 3A shows the effect of K21 on zebrafish egg fertilization. Figure 3B,C show the effects of K21 on zebrafish hatching and growth in the embryonic, larval and adult stages. Figure 3D shows the effect of K21 on the overall survival of fish from the first, second and third generations. Figure 3E shows the effect of K21 on the achievement of the adulthood timeline in the first, second and third generations of zebrafish.

### 3.4. Comparative Study of the Effect of K21 on the Brine Shrimp (Artemia sp.) Hatching Process

**Figure 4:**
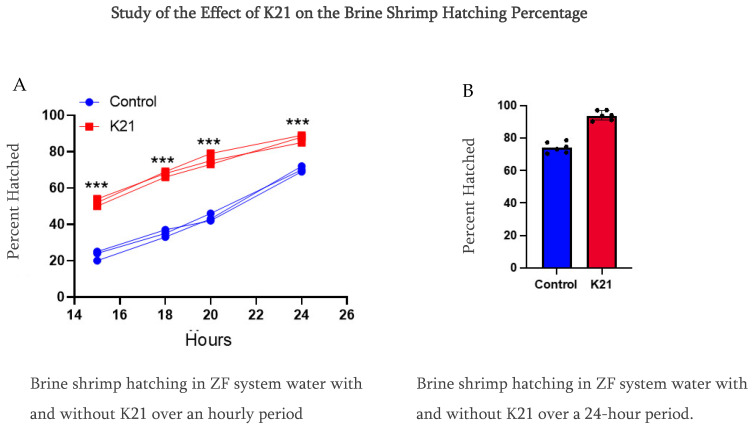
Comparative study of the effect of K21 on the brine shrimp (*Artemia* sp.) hatching process. (**A**) Effect on brine shrimp hatching in control and K21-treated ZF system water at the 15, 18, 20 and 24 h stages. (**B**) Effect of K21 on the brine shrimp hatching process over a 24-h period. (*** *p* < 0.001).

In Experiment 1, we observed that the brine shrimp hatching percentile was over 90% compared to using only ZF system water at 70% at a 24 h hatching time. Furthermore, we reduced the time from 24 h to a 20 h period, and, interestingly, the brine shrimp hatching rate was faster in the K21 group, having the same percentage of over 90%, while in the ZF system water, the brine shrimp hatching rate was approximately 40%. Notably, in this group of only ZF system water, the brine shrimp were under the hatching condition in the egg’s cyst (Figure 4).

In Experiments 2 and 3, we planned to use different water sources, which were RO water and normal tap water, and conducted the brine shrimp hatching experiment for a 24 h time period. We also conducted this experiment using sodium chloride and without sodium chloride. We have observed that the brine shrimp hatching percentage was over 90% as compared to using only RO water and normal tap water in the K21-treated group. While in the group without K21, the percentage was detected to be 70% at 24 h of hatching time using sodium chloride (Figure S1A–D). We have also studied the role of sodium chloride in the hatching of brine shrimp in both water sources with K21 and without the K21 drug. The brine shrimp eggs did not hatch in the absence of sodium chloride under all four conditions. 1. Our findings provide strong evidence of a better hatching ratio of 90% of brine shrimp in the group of treated with the K21 drug as compared to 70% in the group treated without the K21 drug after 24 h of treatment. 2. The K21 drug helps the early hatching process, as observed by the 90% hatching rate in 20 h treatment K21 group while in the group without K21, only 40% of brine shrimps hatched. 3. We have tested multiple water sources using RO water and normal tap water for a 24 h treatment period, and the finding supports the results of Experiment 1. 4. We have also conducted an experiment on the role of sodium chloride in the hatching process and observed that the brine shrimp hatched only in the presence of sodium chloride, while in the group without sodium chloride, the brine shrimp egg remain unhatched.

### 3.5. Histological Evaluation of K21-Treated Adult Zebrafish

**Figure 5:**
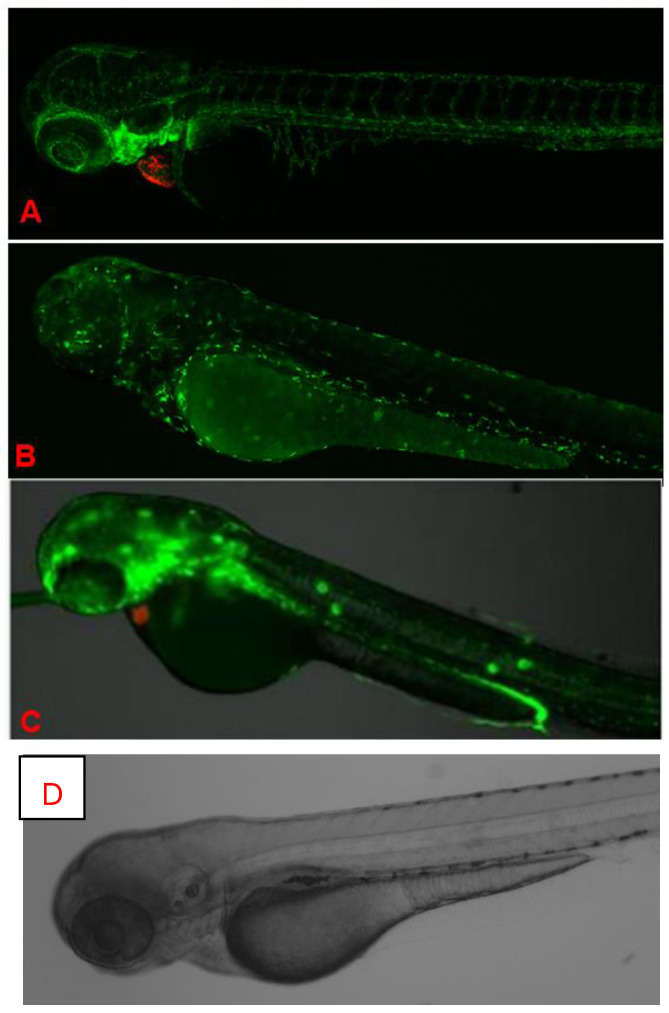
In vivo fluorescence imaging of 72 hpf zebrafish larvae expressing transgenic fluorescent reporters. (**A**). CLSM images of Casper Tg (myl7:RFP; A5:YSP) zebrafish larvae at 72 h hpf. Red fluorescent protein (RFP) expression is driven by the *myl7* promoter, marking cardiomyocytes and confirming heart-specific transgene expression. CLSM images of Casper Tg(Fli:GFP) zebrafish larvae at 72 hpf. GFP expression highlights blood vessels, while RFP marks cardiac tissue, allowing the visualization of cardiovascular structures in the transparent zebrafish. (**B**). CLSM images of Casper Tg(mpeg1:GFP) zebrafish larvae at 72 hpf. GFP expression labels macrophages, with dorsal (upper) and lateral (lower) views showing the microglial distribution and immune cell activity. (**C**). Fluorescence imaging of Casper *Tg(NF-kB:GFP; myl7:RFP*) zebrafish larvae. GFP fluorescence indicates *NF-κB*-driven inflammatory activation, while RFP marks the cardiac region. (**D**). Control image. No abnormalities were detected during K21 treatment in fish progenies from the transgenic lines, and they expressed the normal pattern of fish development.

A histological analysis of adult zebrafish treated chronically with K21 was performed to examine potential changes in the tissue structure and integrity. Hematoxylin and eosin-stained sections from the anterior (heart region; Figure 5A) and posterior (abdominal region; Figure 5B) areas of adult zebrafish were evaluated microscopically at multiple magnifications (2×, 5×, 10×, and 20×).

An examination of the heart region in adult zebrafish revealed intact myocardial structures with clearly defined cardiomyocytes arranged regularly around the heart chambers. There were no evident morphological abnormalities or signs of cardiotoxicity in the treated fish compared to controls. Similarly, histological sections of the abdominal region demonstrated a normal architecture and cellular integrity of internal organs such as the liver, gastrointestinal tract, and surrounding musculature, with no apparent pathological features or inflammatory cell infiltration observed.

Overall, these histological findings indicate that chronic exposure to K21 at the established optimal concentration (1 µL/L) does not adversely impact organ morphology or tissue integrity. Instead, the maintained histological integrity of essential organs, combined with the observed growth enhancement at macroscopic levels, supports the safety and potential physiological benefits of K21 treatment. This further underscores the suitability of K21 as a candidate for biomedical applications and growth enhancement strategies in aquaculture.

### 3.6. Growth and Development of Casper Transgenic Strains in the Presence of K21

To systematically evaluate the potential role of K21, multiple transgenic lines were maintained and studied, including (a) Casper (transparent skin mutant), (b) Casper Tg(myl7:RFP; secA5:YFP; fli:GFP), a transgenic line used for analyzing cardiac function, apoptosis, and vascular function; (c) Casper Tg(myl7:RFP; A5:YFP; NFkB:GFP), used to study inflammatory responses; and (d) Casper Tg(mpeg1:GFP), utilized for examining CNS microglial function. A similar growth-promoting trend was observed in various transgenic and mutant zebrafish strains maintained on the transparent Casper background. All these transgenic lines demonstrated a similar positive trend of enhanced growth and were continuously maintained to facilitate a detailed evaluation of the effects of K21 across multiple generations. The images in Figure 5 show no abnormalities detected during K21 treatment in the fish progenies from the transgenic lines, and they expressed the normal pattern of fish development.

**Figure 6:**
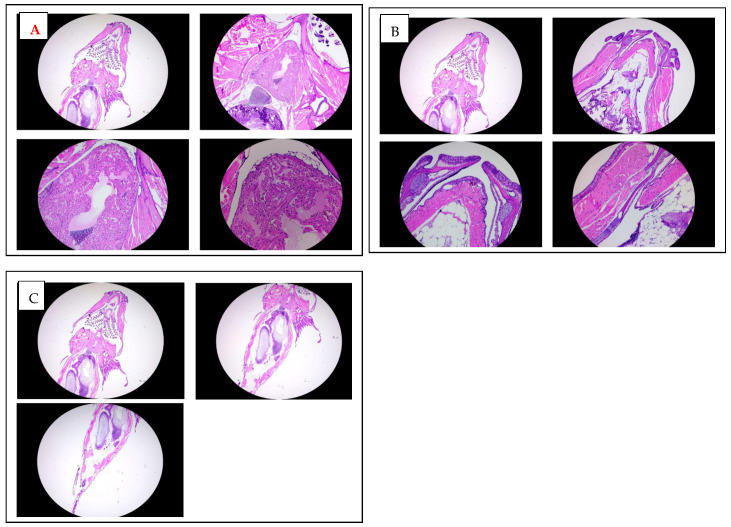
Histological analysis of adult zebrafish treated with K21. (**A**). Hematoxylin and eosin (H&E) staining of transverse sections through the anterior heart region of K21-treated adult zebrafish at increasing magnifications (2×, 5×, 10×, and 20×). The cardiac chambers surrounding myocardial tissue, and adjacent vasculature appear structurally intact, with no signs of fibrosis, inflammation, or pathological remodeling. (**B**). H&E staining of transverse sections of the mouth and jaw area up to trunk region of K21-treated adult zebrafish at 2×, 5×, 10×, and 20× magnifications. (**C**) H&E staining of transverse sections through the posterior abdominal region to the tail area of K21-treated adult zebrafish at 2×, 5×, 10×. The abdominal cavities, including the digestive tract, musculature, and associated organs, exhibit a normal histological architecture with no apparent abnormalities or inflammatory cell infiltration. These findings indicate that chronic K21 exposure does not induce histopathological alterations in the heart or abdominal organs, supporting its safety in long-term applications.

In vivo fluorescence images of different transgenic zebrafish larvae after exposure to K21 are illustrated in Figure 6. Figure 6A presents CLSM images at the 72 hpf stage of homozygous larvae from the transgenic zebrafish line Casper Tg(myl7:RFP; secA5:YFP;Fli:GFP). RFP specifically indicates cardiomyocyte expression through the myl7 promoter for highlighting cardiac development and function. No developmental anomalies or malformations were noted in these larvae, suggesting normal cardiac morphology and health following K21 treatment. GFP clearly visualizes vascular structures, while RFP delineates heart-specific myl7 expression. The vasculature appears structurally intact, without abnormalities, supporting the safety and beneficial effects of K21 on vascular and cardiac development in transgenic models.

Figure 6B presents CLSM images of the 72 hpf stage of Casper Tg(mpeg1:GFP) zebrafish larvae. This transgenic line allows for the visualization of macrophage and microglia populations marked by GFP expression. Imaging from dorsal and lateral views revealed robust GFP-positive immune cells that suggest a normal microglial cell distribution and function under K21 treatment conditions. Figure 6C presents CLSM images Casper Tg(NF-κB:GFP; myl7:RFP) larvae. GFP fluorescence specifically indicates NF-kB activation related to inflammation, while RFP expression indicates cardiac structures. The images showed controlled, minimal NF-kB activity. The findings suggest that K21 may suppress unnecessary inflammatory responses. Clear expression patterns of RFP indicate healthy cardiac structures in these transgenic strains, further supporting K21’s potential anti-inflammatory benefits without cardiac side effects.

### 3.7. Anti-Inflammatory Effects of K21 on LPS-Treated NF-κB/Casper Transgenic Zebrafish

NF-κB signaling regulates cell survival, tissue growth, and proliferation and is a major mediator of inflammation [32]. This pathway controls the expression of multiple genes involved in inflammatory responses [33]. Elevated NF-κB activity has been associated with increased mortality, particularly in individuals with cancer and cardiovascular diseases [34]. Hence, a Casper NF-κB:GFP transparent transgenic zebrafish model was developed to study inflammatory processes in vivo.

**Figure 7:**
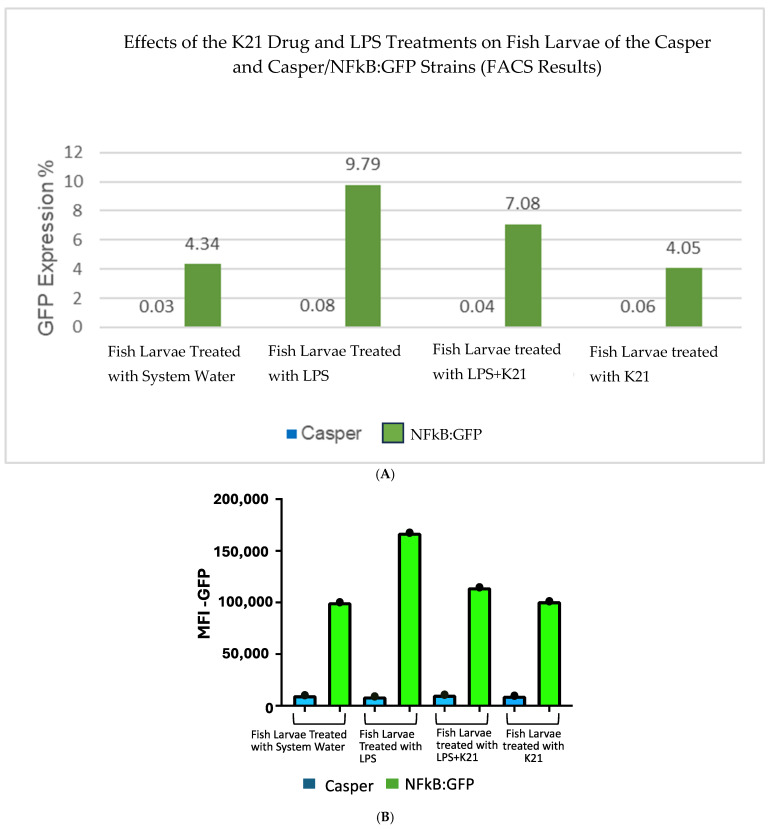
(**A**). Bar chart presenting GFP expression in Casper Tg(NF-κB:GFP) zebrafish larvae under different treatment conditions.Fluorescence-activated cell sorting (FACS) analysis of GFP expression in the zebrafish larvae following different treatments. The LPS-treated group shows the highest GFP expression (9.79%), confirming a significant inflammatory response. The LPS + K21 co-treatment group exhibits a reduction in GFP expression (7.08%), indicating the anti-inflammatory effect of K21. The K21-only treatment group (4.05%) shows GFP levels comparable to the control group (4.34%), demonstrating that K21 does not induce inflammation on its own. These results support the role of K21 as a modulator of inflammation, effectively reducing LPS-induced NF-κB activation in zebrafish larvae. (**B**). Bar chart presenting the AFI of GFP expression in Casper Tg(NF-κB:GFP) zebrafish larvae along with corresponding non-transgenic control Casper zebrafish larvae under different treatment conditions. Fluorescence-activated cell sorting (FACS) analysis of GFP expression in the zebrafish larvae following different treatments. Blue bar show the Casper line and green bars show GFP expression.

**Figure 8:**
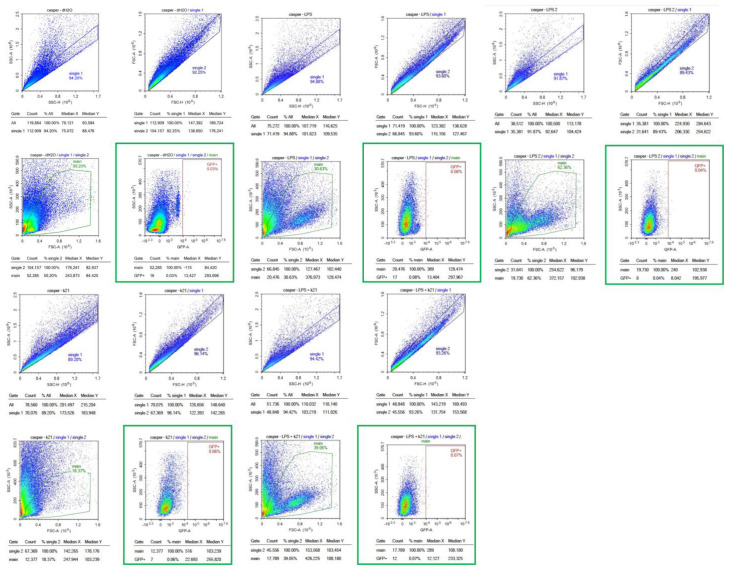

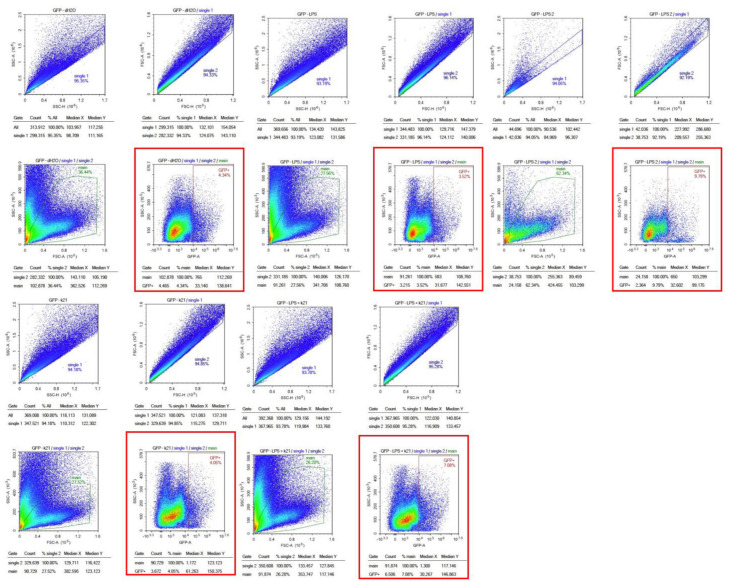
Flow cytometry analysis of homogenized Casper Tg(NF-κB:GFP) zebrafish larvae under different treatment conditions. Scatter plots of homogenized samples of zebrafish larvae under four experimental conditions: control (untreated), K21 treatment, LPS-induced inflammation, and LPS + K21 co-treatment. GFP expression indicates NF-κB activation as a marker of the inflammatory response.

The effect of the K21 treatment on NF-κB activation in the Casper NF-κB:GFP zebrafish larvae was quantitatively assessed using flow cytometry. Scatter plots of homogenized larval samples from Casper/NF-kB:GFP transgenic zebrafish were generated, comparing four experimental conditions: control (system water), LPS treatment, LPS combined with K21, and K21 alone (Figure 7). GFP-positive populations, indicative of NF-κB activation, were clearly distinguished and quantified for each treatment condition. GFP expression was significantly increased in larvae exposed to LPS (9.79 ± 0.08%) compared to controls (4.34 ± 0.03%). Notably, larvae treated simultaneously with LPS and K21 exhibited decreased numbers of GFP-positive cells (7.08 ± 0.04%), suggesting a reduction in inflammation. Larvae exposed only to K21 showed GFP expression (4.05 ± 0.05%) comparable to the control, confirming the absence of pro-inflammatory effects of K21 alone (Figure 8). Collectively, the flow cytometry results indicate that K21 effectively reduces NF-κB-mediated inflammation triggered by LPS exposure.

Non-transgenic Casper mutant larvae were included as a control to rule out any artifacts or false observations. No GFP expression was detected in any of the counterpart groups. These findings establish K21 as a potential anti-inflammatory agent for LPS-induced inflammation and highlight its inhibitory effects on NF-κB-mediated inflammatory signaling. The results provide insights into the therapeutic potential of K21 in experimental treatment modalities targeting NF-κB-related inflammation, particularly in the context of cardio-oncology.

### 3.8. Genomic Analysis of K21-Treated Zebrafish: Differential Gene Expression

**Figure 9:**
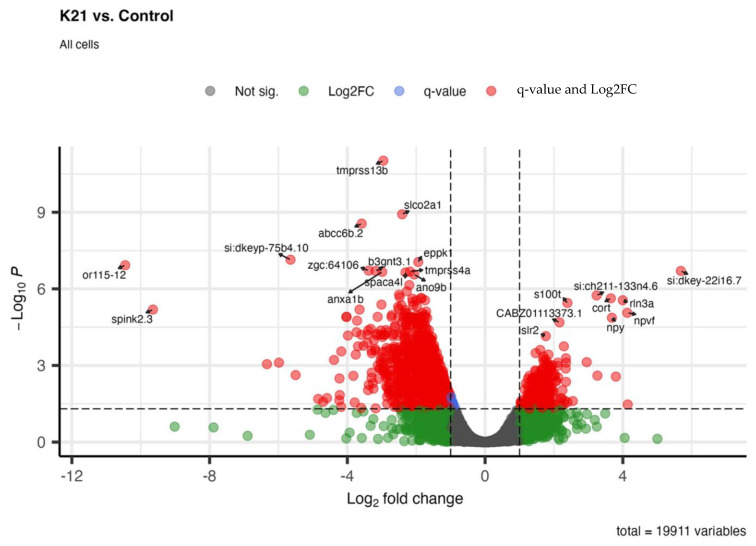
Whole-organism transcriptomic analysis of K21-treated vs. control zebrafish larvae. Volcano plot displaying differentially expressed genes (DEGs) in K21-treated zebrafish larvae compared to controls. Each point represents a gene, plotted based as the log_2_ fold change (*x*-axis) and statistical significance (−log₁₀ *p*-value, *y*-axis). Genes that were significantly upregulated (red) and downregulated (green) are highlighted, while non-significant changes appear in gray.

Figure 9 shows the differential gene expression analysis comparing zebrafish larvae treated with K21 against untreated controls. In the volcano plot, significantly upregulated genes (red dots on the right side) and downregulated genes (red dots on the left side) are visualized based on their statistical significance (adjusted *p*-value < 0.05) and magnitude of log_2_ fold change. Prominent upregulated genes identified include those involved in neuronal differentiation (e.g., *rln3a*), neuropeptide signaling (e.g., *npy* and *npvf*), and calcium-binding proteins (*s100t*). The upregulation of these genes suggests an enhancement of neuronal development and function by K21. Conversely, significantly downregulated genes (left side of the plot) included genes involved in protease inhibition (*spink2.3*), membrane transport (*tmprss13b* and *slco2a1*), and ion transport. The downregulation of these genes suggests the possible suppression of pathways associated with inflammation, apoptosis, or stress responses.

Overall, the gene expression profile illustrates that K21 influences major biological processes, notably promoting neuronal growth and development pathways while suppressing inflammatory, apoptotic, and stress-related genes. These molecular findings align closely with observed phenotypic outcomes, indicating a beneficial impact of K21 on zebrafish physiology and providing mechanistic insights into its growth-promoting and anti-inflammatory properties.

**Figure 10:**
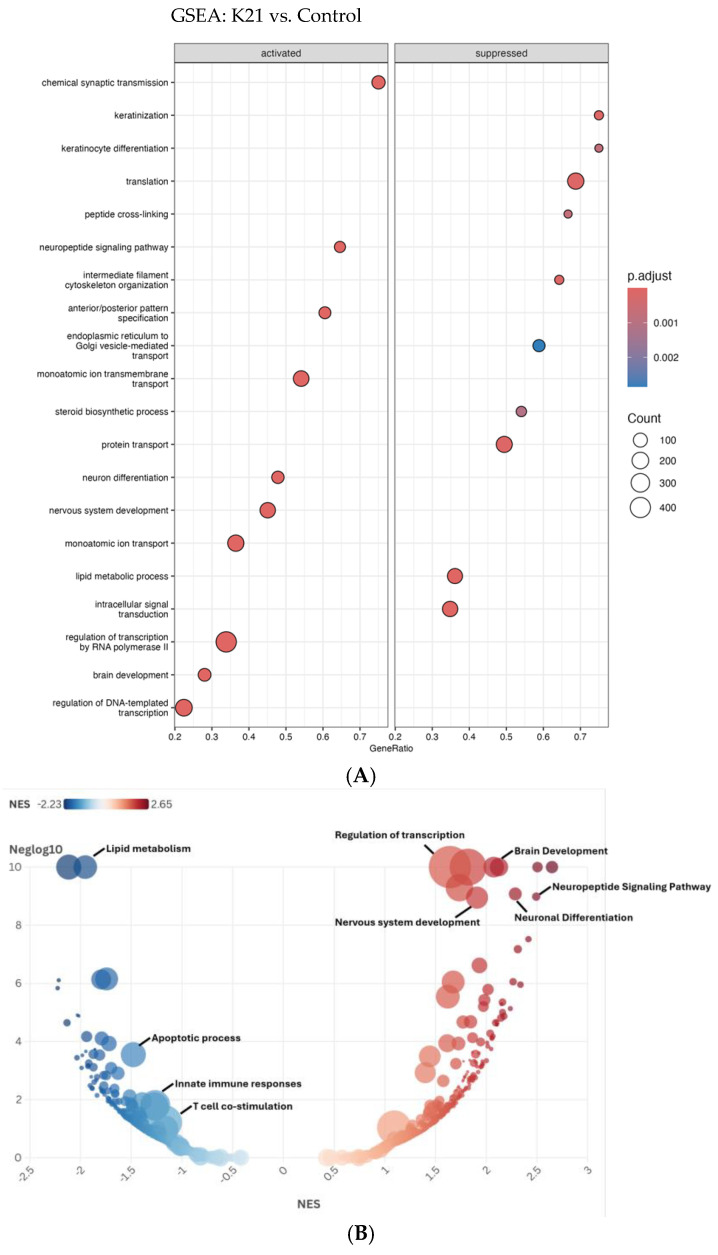
Gene Set Enrichment Analysis (GSEA) of biological pathways influenced by the K21 treatment. (**A**). The dot plot displays enriched biological pathways in K21-treated zebrafish larvae compared to controls. Pathways on the left represent activated processes, while those on the right are suppressed. The gene ratio (*x*-axis) reflects the proportion of differentially expressed genes involved in each pathway. The size of the dots indicates the number of genes in the pathway, and the color gradient represents statistical significance (*p.adjust*), with darker red indicating higher significance. (**B**). The bubble plot provides a normalized enrichment score (NES) ranking of pathways, with positively enriched (red) pathways. The *y*-axis represents −log10 (*p*-value), emphasizing the statistical strength of each pathway.

The Gene Set Enrichment Analysis (GSEA) depicted in the dot plot (Figure 10A) provides comprehensive insights into the biological pathways activated or suppressed by K21 treatment in zebrafish larvae. The pathways shown are ranked by GeneRatio, which indicates the proportion of significantly altered genes within each pathway, while the dot size represents the number of genes (count) involved, and the color intensity indicates the adjusted *p*-value (statistical significance).

Among the activated pathways, “chemical synaptic transmission” showed the highest GeneRatio, indicating a strong activation of neuronal communication processes. Additional significantly activated pathways include “regulation of DNA-templated transcription”, “brain development”, “nervous system development”, and “neuronal differentiation”, suggesting that K21 positively influences neuronal growth, differentiation, and brain developmental processes. Pathways related to “lipid metabolic process”, “monoatomic ion transport”, and “protein transport” were also significantly enriched, highlighting the potential metabolic and ion homeostasis regulatory effects of K21.

Conversely, pathways suppressed by K21 treatment predominantly involved processes associated with cellular stress and immune responses. Notably suppressed pathways included “translation”, “peptide cross-linking”, “monoatomic ion transmembrane transport”, and pathways related to intracellular protein transport and vesicle-mediated trafficking between the endoplasmic reticulum and Golgi apparatus. The significant downregulation of pathways associated with fundamental cellular biosynthetic processes (“translation” and “steroid biosynthetic process”) suggests a selective suppression by K21 that potentially redirects cellular resources toward growth-promoting pathways.

The bubble plot (Figure 10B) further refines and visually confirms the GSEA results obtained from the dot plot analysis, emphasizing pathways enriched by the K21 treatment through the distribution of normalized enrichment scores (NESs). Pathways activated by K21 (depicted in red) demonstrate significant enrichment for biological processes related to neuronal and brain development. The most prominently activated pathways included “brain development”, “neuronal differentiation”, “neuropeptide signaling pathway”, and “nervous system development”, all showing notably high NESs and statistical significance. These findings strongly support the hypothesis that K21 positively influences neuronal growth and functional maturation, potentially via enhancing synaptic communication and neuronal differentiation processes.

Suppressed pathways (depicted in blue) showed strong enrichment for biological processes involved in the immune response and cellular stress. Notably, “apoptotic process”, “innate immune responses”, and “T-cell co-stimulation” were significantly downregulated, reinforcing the evidence of K21’s potential anti-inflammatory and anti-apoptotic effects. The downregulation of pathways related to “lipid metabolism” also suggests a metabolic reprogramming by K21 treatment, possibly indicative of reduced inflammatory metabolic signaling or stress responses.

The clear delineation between the strongly enriched activated pathways and distinctly suppressed pathways provides robust evidence for the targeted regulatory effects of K21. The activation of neuronal developmental processes coupled with the suppression of inflammation and apoptosis provides a coherent molecular explanation for the observed improvement in zebrafish growth and overall health upon K21 exposure.

## 4. Conclusions

The present study comprehensively examined the effects of exposure to the antimicrobial agent K21 on zebrafish growth, development, and physiological responses across multiple generations. The findings from this study demonstrate that K21 significantly enhances zebrafish growth, as evidenced by the increased body mass, improved hatching rates, and accelerated larval and juvenile development, without adverse effects on tissue integrity or morphology. Histological evaluations further confirmed these observations, showing that short-term and long-term K21 exposure at the optimal dosage (1 µg/L) maintained structural integrity in vital organs, including the heart and abdominal tissues, without signs or symptoms of toxicity or morphological abnormalities. K21 helps the fish grow to larger sizes than control fish without organ malformations

Flow cytometry analyses utilizing the transgenic Casper Tg(NF-κB:GFP) zebrafish model demonstrated that K21 possesses notable anti-inflammatory properties. Specifically, K21 effectively attenuated lipopolysaccharide-induced inflammation by reducing NF-κB pathway activation, as quantified by a reduction in GFP expression levels. Complementary single-cell RNA sequencing and subsequent genomic analyses revealed molecular insights into these phenotypic improvements. The results showed the significant upregulation of genes linked to neuronal differentiation, neuropeptide signaling, and brain development, alongside the suppression of apoptotic and immune activation pathways. This transcriptional shift supports the potential role of K21 in neuronal development, metabolic regulation, and immune modulation.

Collectively, the integrative approach combining phenotypic, histological, and genomic data underscores the promise of K21 as a candidate therapeutic agent for inflammation-related conditions and growth enhancement. Future studies should focus on exploring the detailed mechanistic interactions at cellular and molecular levels and validating these promising observations in large animal models. This will help advance the translational potential of K21 into broader biomedical and clinical settings.

Our findings provide strong evidence of better hatching ratios of 90% of brine shrimp in the group treated with the K21 drug as compared to 70% in the group treated without the K21 drug in a 24 h treatment; the K21 drug helps the early hatching process, as observed by the 90% hatching rate in 20 h treatment K21 group, while in the group without K21, only 40% of brine shrimps hatched.

## Figures and Tables

**Table 1:** Comparison of the growth rates between untreated control and K21-treated zebrafish populations.

SN	Stage	Normal Control: No K21 Treatment	K21-Treated Fish Progeny
F1 and F2 generation zebrafish fish progenies			
1	Zebrafish egg production	Normal egg laying production	Improved egg laying pattern
2	Zebrafish egg fertilization	10–20 eggs unfertilized	The egg fertilization percentile improved to 90–95%
3	Embryonic hatching pattern	Over 20% embryos were unhatched	The unhatched embryo percentile was reduced by 5%
4	Embryonic growth: hatching pattern at the one-day-old stage	Embryo growth and hatching patterns are normal; embryos embedded in chorion	Improved embryonic growth and hatching process; 10% embryos hatched into larvae by coming out from the chorion
5	Embryonic growth: hatching pattern at the two-day-old stage	Embryo growth and hatching patterns are normal; 10% of embryos hatched into the larval stage by coming out from the chorion	Improved embryonic growth and hatching process; over 40% embryos hatched into larvae by coming out from the chorion
6	Embryonic growth: hatching pattern at three-day-old stage	Embryo growth and hatching patterns are normal; 80% of embryos hatched into the larval stage by coming out from the chorion and were yet to perform swimming	Improved embryonic growth and hatching; 100% of embryos hatched into larvae and started to swim
**Growth of F2 generation zebrafish progenies**			
1	Growth into adulthood	The normal zebrafish growth pattern requires 90 days to reach adulthood, at which point they are considered breeders. Mature zebrafish develop a bulky belly region due to the accumulation of eggs, indicating reproductive readiness.	The growth pattern in 70-day-old K21-treated zebrafish was visibly accelerated, as indicated by the clear distinction between males and females, with females exhibiting a bulky belly region due to egg accumulation.
2	Breeding set up of second-generation progeny	Three-month-old zebrafish were considered ready for breeding to produce the next generation.	Seventy-five-day-old zebrafish progeny were paired as breeders, with males and females arranged for breeding in six batches. Out of the six batches, four successfully laid eggs. The eggs were healthy, with over 90% fertilization occurring within 30 min of laying. After fertilization, embryonic development began at the zygote stage.
**Growth of F3 Generation Zebrafish Progenies**			
1	Third-generation K21-treated progeny	Three-month-old zebrafish were considered ready for breeding to produce the next generation.	The third-generation progeny of the K21-treated F1 and F2 groups exhibited an improved growth and development pattern. This generation was maintained as the next K21-treated strain. The third-generation fish population reached adulthood within 75 days and successfully entered the breeding process to produce the next generation.

## Data Availability

The original contributions presented in this study are included in the article/Supplementary Materials. Further inquiries can be directed to the corresponding author.

## Supplementary Materials

The following supporting information can be downloaded at https://www.mdpi.com/article/10.3390/biomedicines13061267/s1: Figure S1. Comparison of Artemia (brine shrimp) hatching in K21-treated ZF system water (S1A) hatching with hatching in zebrafish system water without K21 (S1B). Brine shrimp hatched to the swimming stage (10× Image; S1C), brine shrimp hatched to the swimming stage (5× image; S1D).
