# Supplementary material for: Evaluation of the Effects of the Quaternary Ammonium Silane K21 on Zebrafish Viability, Toxicity, Growth, and Development
Source: Biomedicines. 2025 May 22;13(6):1267. doi: 10.3390/biomedicines13061267 (PMC12189560; doi:10.3390/biomedicines13061267)
Supplement: Supplementary file 1 [file biomedicines-13-01267-s001.zip › biomedicines-3592283-supplementary.DOC]

**Rajpurohit et al Manuscript ID biomedicines-3592283 V2 Supplement Figures**

Evaluation of The Effects of the Quaternary Ammonium Silane K21 on the Zebrafish Viability, Toxicity Growth and Development

Authors Listed : Surendra K Rajpurohit et al

**Supplement Figure 1**


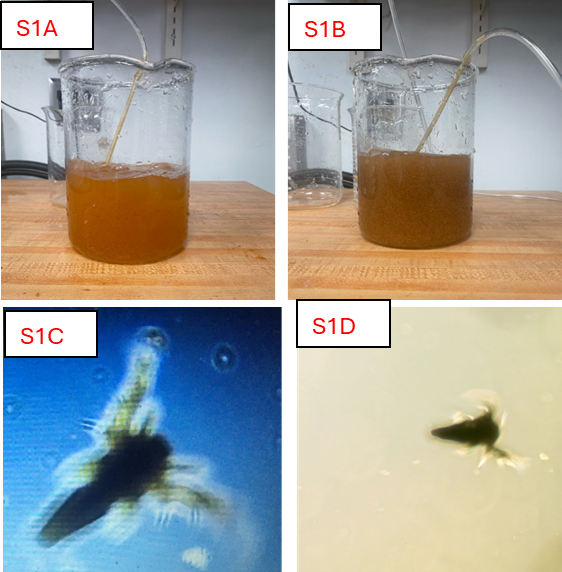


**Supplement Figure S1**. Artemia (Brine shrimp) Hatching in K21 treated ZF system water (S1A) hatching comparison with hatching in zebrafish system water without K21 (S1B). Brine shrimp hatched swimming stage (10x Image; S1C), brine shrimp hatched swimming stage (5x image; S1D).
